# Cuproptosis-related gene SLC31A1: prognosis values and potential biological functions in cancer

**DOI:** 10.1038/s41598-023-44681-8

**Published:** 2023-10-18

**Authors:** Yue Qi, Qingqing Yao, Xuanyan Li, Xinyu Li, Wenwen Zhang, Pengpeng Qu

**Affiliations:** 1 Clinical School of Obstetrics and Gynecology Center, Tianjin Medical University, Tianjin, China; 2 Department of Gynecological Oncology, Tianjin Central Hospital Gynecology Obstetrics, No. 156, Nansanma Road, Nankai District, Tianjin, 300000 China; 3 Nankai University School of Medicine, Nankai University, Tianjin, China

**Keywords:** Cancer, Computational biology and bioinformatics

## Abstract

Cuproptosis is a unique type of cell death that may influence tumour formation by targeting lipoylated tricarboxylic acid cycle proteins. Solute carrier family 31 member 1 (SLC31A1), an important copper transporter, influences dietary copper absorption in the cell membrane. However, various SLC31A1 properties in pan-cancer profiles remain unknown. This study investigated the role of SLC31A1 in human malignancies and analysed its prognostic value. Raw data were obtained from The Cancer Genome Atlas database and processed using numerous internet databases, including UALCAN, GEPIA, cBioPortal, TIMER2.0, and Human Protein Atlas. SLC31A1 expression was found to be elevated in cervical, endometrial, and breast cancers compared to that in normal tissues, but reduced in clear cell renal cell carcinoma, liver hepatocellular carcinoma, and lung adenocarcinoma. Furthermore, SLC31A1 expression was strongly associated with overall survival and disease-free survival in several cancers. SLC31A1 gene mutations and methylations were identified in 33 cancers. SLC31A1 expression was positively correlated with immune cells in immune infiltration data. Single-cell sequencing revealed that SLC31A1 may play key roles in DNA repair, DNA damage, and proliferation. These findings may lead to better understanding of SLC31A1 in pan-cancer profiles and suggest that SLC31A1 could be a viable predictive biomarker, particularly in gynaecological cancers.

## Introduction

Cancer is a prominent cause of illness and mortality worldwide; therefore, it is one of the most pressing public health challenges. According to the American Cancer Society, cancer is expected to cause 609,360 deaths and 1,918,030 new cases in the United States by 2022, according to the American Cancer Society^1^. Despite recent breakthroughs in detection and treatment choices, individuals with advanced stage cancers continue to have poor prognoses with exceptionally high recurrence rates. Therefore, enhancing prognostic predictions and identifying new treatment targets are critical for public health.

Copper is a trace element that serves as a cofactor for more than thirty enzymes involved in a wide range of physiological processes. Recent research has shown that cells maintain copper at a low concentration, and that increasing the cumulative concentration of copper results in cytotoxicity and even death^2^. Furthermore, the dysregulation of copper homeostasis may contribute to tumour metastasis^3^. Tsvetkov recently proposed that cuproptosis, a novel type of cell death, contributes to cancer progression and metastasis^4^. Thirteen cuproptosis-related genes were identified, including FDX1, SLC31A1 (solute carrier family 31 member 1), LIPT1, LIAS, DLD, DBT, GCSH, DLST, DLAT, PDHA1, PDHB, ATP7A, and ATP7B. Of these, SLC31A1 influences the uptake of dietary copper via copper transporters located in the cell membrane. Interestingly, an increasing number of studies have found that dysregulated copper levels are associated with various cancers, including lung, breast, kidney, and colorectal cancers. Given the importance of SLC31A1 in copper homeostasis, it is necessary to investigate its specific roles of SLC31A1 in various cancers.

We used multiple methods common in the field of bioinformatics to investigate the molecular mechanisms of SLC31A1 and provide comprehensive pan-cancer genomics and clinical prognoses. We examined the relationship between SLC31A1 expression and patient survival using datasets obtained from the Cancer Genome Atlas (TCGA) and Gene Expression Omnibus databases (GEO). Gene enrichment and immune cell infiltration were investigated to better understand the underlying molecular mechanisms and potential benefits.

## Results

### Differing expression of SLC31A1 and correlation analysis in human pan-cancer

**Figure 1:**
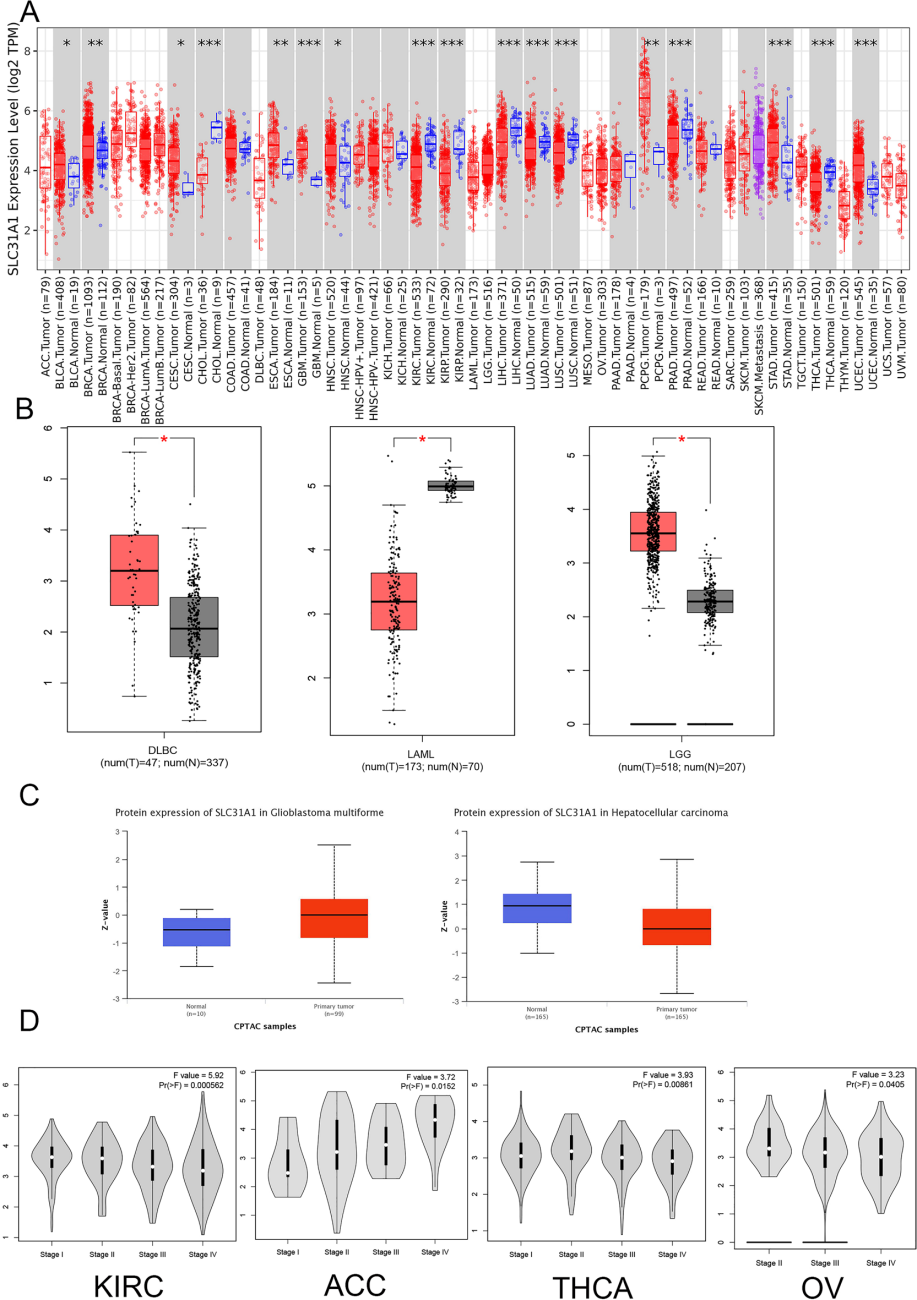
SLC31A1 expression in pan-cancer profile. (**A**) The expression of SLC31A1 in different tumors in TIMER2.0. (**B**) The expression of SLC31A1 from TCGA and GTEx. (**C**) The expression of SLC31A1 at protein levels of GBM and LIHC in CPTAC. (D)The relationship of SLC31A1 expression to TNM stage in KIRC, ACC, THCA and OV. (*, p < 0.05; **, p < 0.01; ***, p < 0.001, ns: no statistical differences).

Using TIMER2.0^5^, we first observed the expression of SLC31A1 in normal and malignant cancer tissues. As depicted in Fig. 1A, the expression of SLC31A1 is significantly lower in certain tumours than in the adjacent normal tissues, including cholangiocarcinoma (CHOL), kidney renal clear cell carcinoma (KIRC), kidney renal papillary cell carcinoma (KIRP), liver hepatocellular carcinoma (LIHC), lung adenocarcinoma (LUAD), lung squamous cell carcinoma (LUSC), thyroid carcinoma (THCA), and prostate adenocarcinoma (PRAD). In contrast, SLC31A1 expression is significantly upregulated in certain tumours such as those of bladder urothelial carcinoma (BLCA), breast invasive carcinoma (BRCA), cervical squamous cell carcinoma, endocervical adenocarcinoma (CESC), oesophageal carcinoma (ESCA), glioblastoma multiforme (GBM), head and neck squamous cell carcinoma (HNSC), and uterine corpus endometrial carcinoma (UCEC). Additionally, the expression of SLC31A1 in other malignancies, including colon adenocarcinoma (COAD), kidney chromophobe (KICH), pancreatic adenocarcinoma (PAAD), and rectal adenocarcinoma (REC), was insignificant. We further analysed the expression of SLC31A1 using TCGA and Genotype-Tissue Expression (GTEx) datasets because certain normal tissue data were unavailable. As shown in Fig. 1B, SLC31A1 expression is upregulated in lymphoid neoplasm, diffuse large B-cell lymphoma (DLBC), and cerebral low-grade leukaemia (LGG), but downregulated in acute myeloid leukaemia (LAML) and no significant difference in expression was found with other malignancies (Fig. S1).

The total protein expression of SLC31A1 was found to be upregulated in GBM and downregulated in LIHC, per the Clinical Proteomic Tumour Analysis Consortium (CPTAC)^6^ dataset from the National Cancer Institute (Fig. 1C). Other tumours showed no significant features. In addition, we investigated the relationship between SLC31A1 expression and pathological tumour stage using Gene Expression Profiling Interactive Analysis 2.0(GEPIA2.0)^7^ and found that SLC31A1 expression is associated with KIRC, adrenocortical carcinoma (ACC), ovarian serous cystadenocarcinoma (OV), and THCA (Fig. 1D).

**Figure 2:**
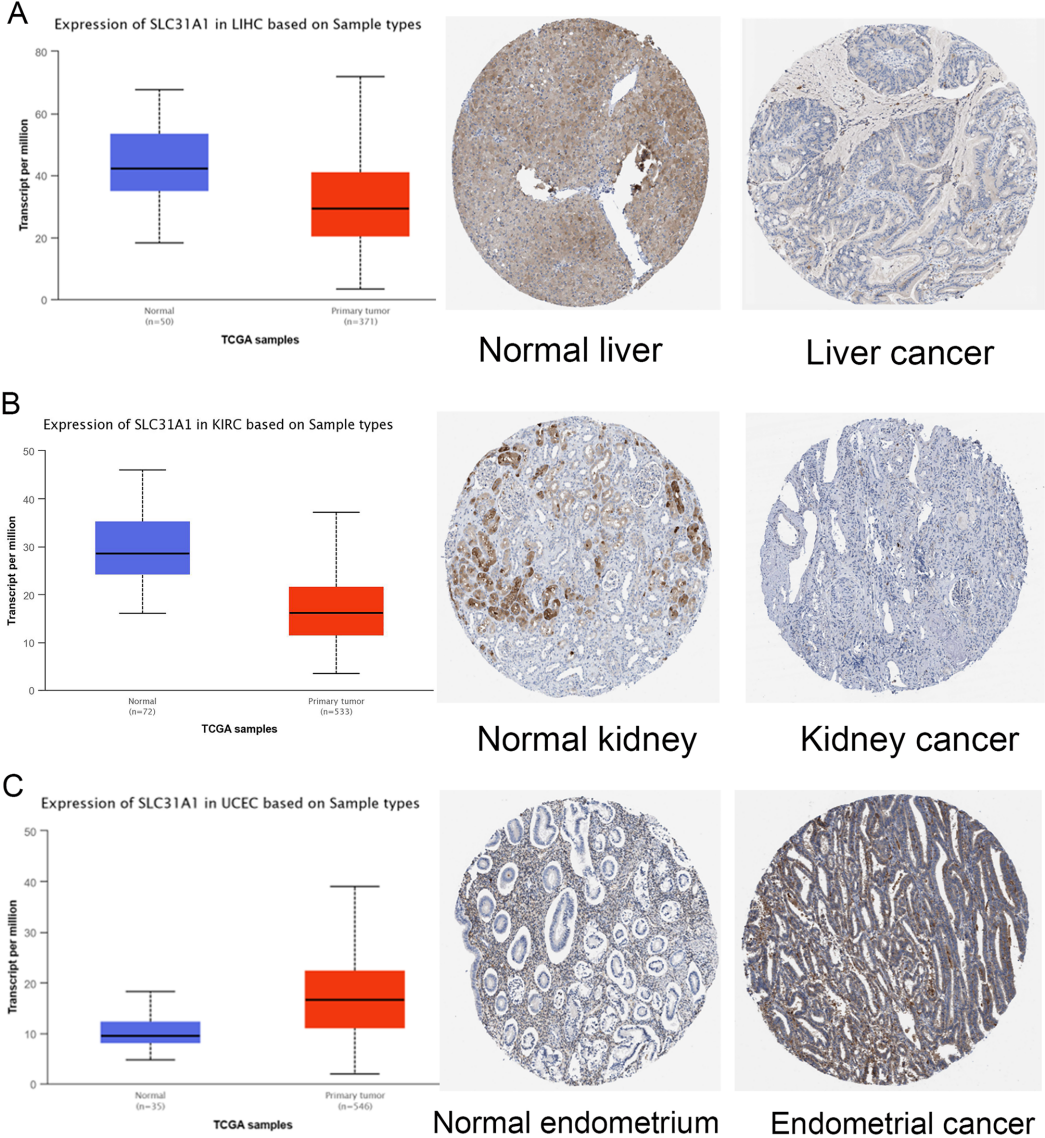
SLC31A1 expression between tumors and adjacent normal tissues. (**A**–**C**) SLC31A1 expression in LIHC, KIRC and UCEC from UALCAN and HPA databases.

We also analysed the expression of SLC31A1 at the mRNA and protein levels using the UALCAN database^8^ and investigated the immunohistochemistry (IHC) results using the Human Protein Atlas (HPA) datasets^9^. SLC31A1 IHC staining is weaker in KIRC and LIHC primary tumour tissues than in normal tissues (Fig. 2A, B), but more positive in UCEC than in the normal endometrium (Fig. 2C). Overall, we discovered substantial differences in SLC31A1 expression among various tumours with distinct profile types. Therefore, we inferred that SLC31A1 plays a crucial role in tumour progression.

### SLC31A1 was related to patients’ prognosis in pan-cancer

**Figure 3:**
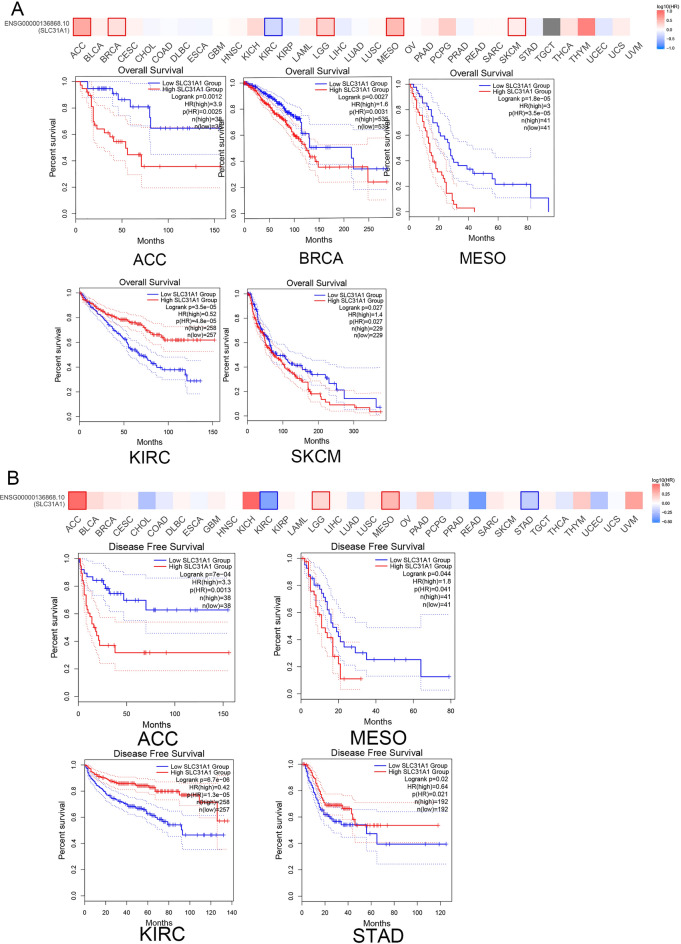
Survival analysis of SLC31A1 in pan-cancer. (**A**, **B**) The relationship between SLC31A1 and patients’ prognosis in GEPIA2.0, including OS (**A**) and DFS (**B**).

To further dissect the pertinent clinical values associated with the cuproptosis-related SLC31A1 gene, we used GEPIA2.0 to analyse patients' overall survival (OS) and disease-free survival (DFS). As shown in Fig. 3A, patients with high SLC31A1 expression have a shorter OS than those with low SLC31A1 expression in cases of ACC (p = 0.0025), BLCA (p = 0.0031), mesothelioma (MESO) (p = 3.5e−05), and skin cutaneous melanoma (SKCM) (p = 0.027). Intriguingly, the low SLC31A1 cohort in the KIRC group had significantly poorer OS (p = 4.8e−05) than those with higher SLC31A1 expression, indicating that SLC31A1 may be a prognostic factor in KIRC. Figure 3B shows that high SLC31A1 expression was associated with DFS in the ACC (p = 0.0013) and MESO (p = 0.04) groups. Conversely, low SLC31A1 expression was associated with a subpar DFS (p = 1.3e−05). These results indicate that SLC31A1 plays a crucial role in patient survival, particularly those with ACC, MESO, and KIRC. In addition, the expression profiles and prognostic values suggest that SLC31A1 functions as a tumour suppressor gene in KIRC.

### Mutation of SLC31A1 in pan-cancer profiles

**Figure 4:**
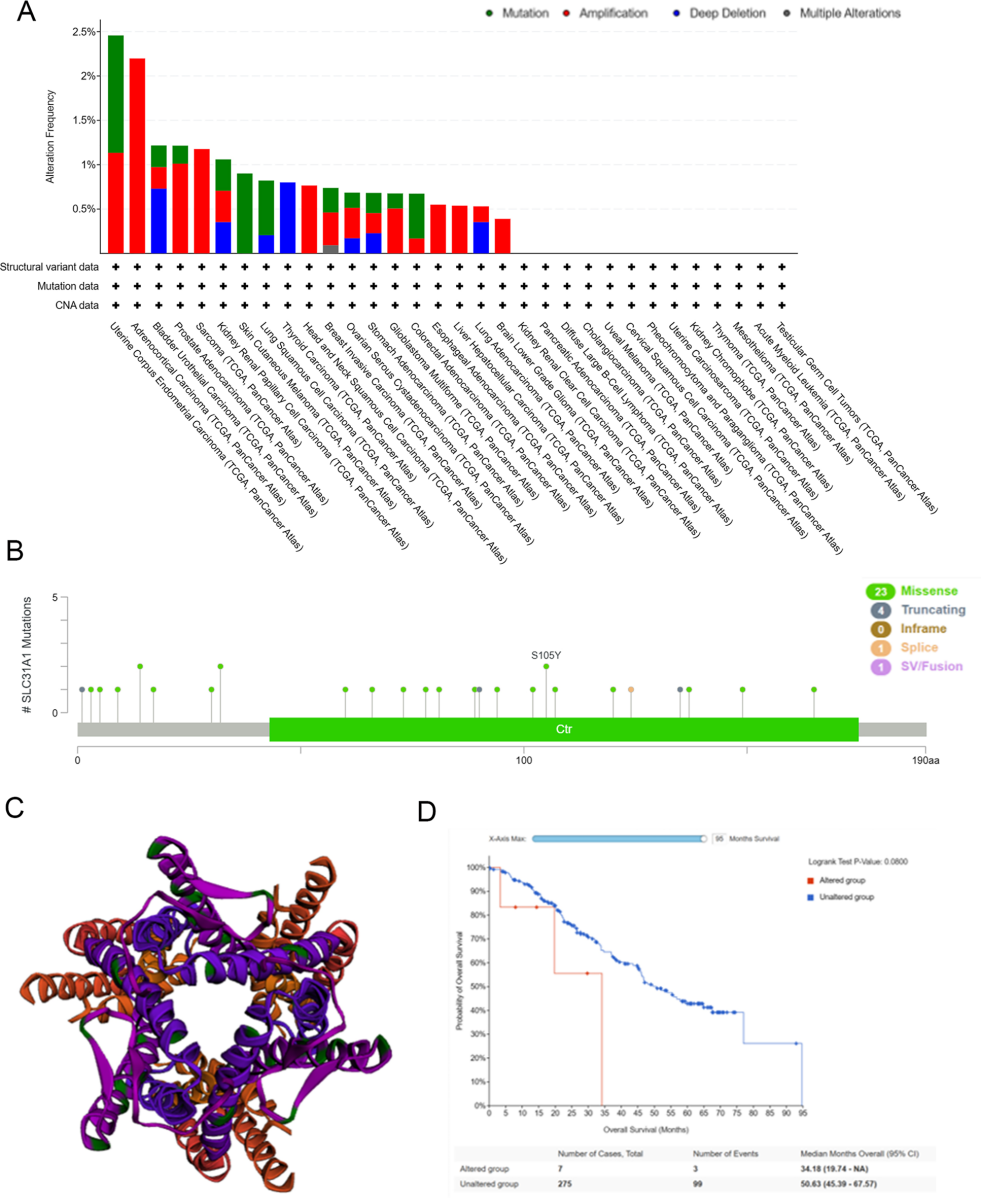
Gene alternations of SLC31A1 in pan-cancer. (**A**) The different mutation types of SLC31A1 in different tumors in cBioPortal database and mutation site (**B**). (**C**) The 3D protein structure of SLC31A1. (**D**) The prognosis relationship between altered group and unaltered group.

To investigate the variation of SLC31A1 in cancer malignancies, we utilised the cBioPortal^10^ platform to analyse mutations in TCGA datasets. As shown in Fig. 4A, UCEC has the highest mutation rate (approximately 2.5%), whereas ACC has the highest amplification rate (> 2%). THCA has the highest frequency of “deep deletions” (at nearly 1%). Missense and truncating mutations constitute the majority of SLC31A1 mutations in different cancers. For example, in patients with GBM, we could detect a missense mutation within the Ctr domain that causes S105Y (Serine converts to Tyrosine) (Fig. 4B). Figure 4C displays the three-dimensional structure of SLC31A1. As for the association between SLC31A1 genetic variations and patients’ survival prognosis, we identified the effect of SLC31A1 genetic variations on patients' overall survival (Fig. 4D); however, further clinical patient data are required to confirm this relationship.

### Promoter methylation of SLC31A1 in cancers

**Figure 5:**
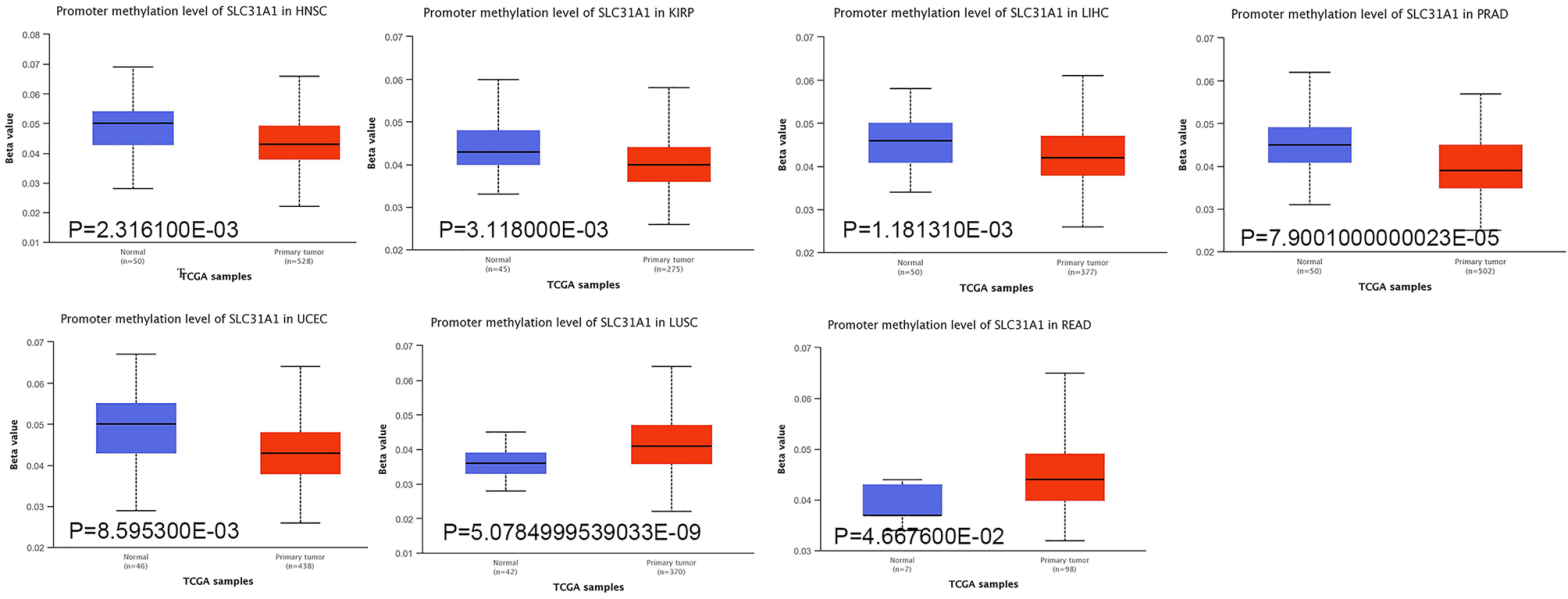
DNA promoter methylation of SLC31A1 in pan-cancer between normal and primary tumor tissues by UALCAN.

Recent studies have demonstrated that promoter methylation is a crucial factor influencing gene transcription and expression and in tumour oncogenesis. Therefore, we analysed the disparities in promoter methylation between cancerous and normal tissues. In several malignancies, including HNSC, KIRP, LIHC, PRAD, and UCEC, SLC31A1 promoter methylation levels are significantly decreased. In contrast, promoter methylation is significantly elevated in LUSC and READ (Fig. 5). In addition, several malignancies are negatively correlated with promoter methylation and tumorigenesis (Fig. S2). These results indicate that SLC31A1 promoter methylation may result in tumour transcription, thereby influencing tumour progression.

### Correlation between SLC31A1 and immune cell infiltration in cancers

**Figure 6:**
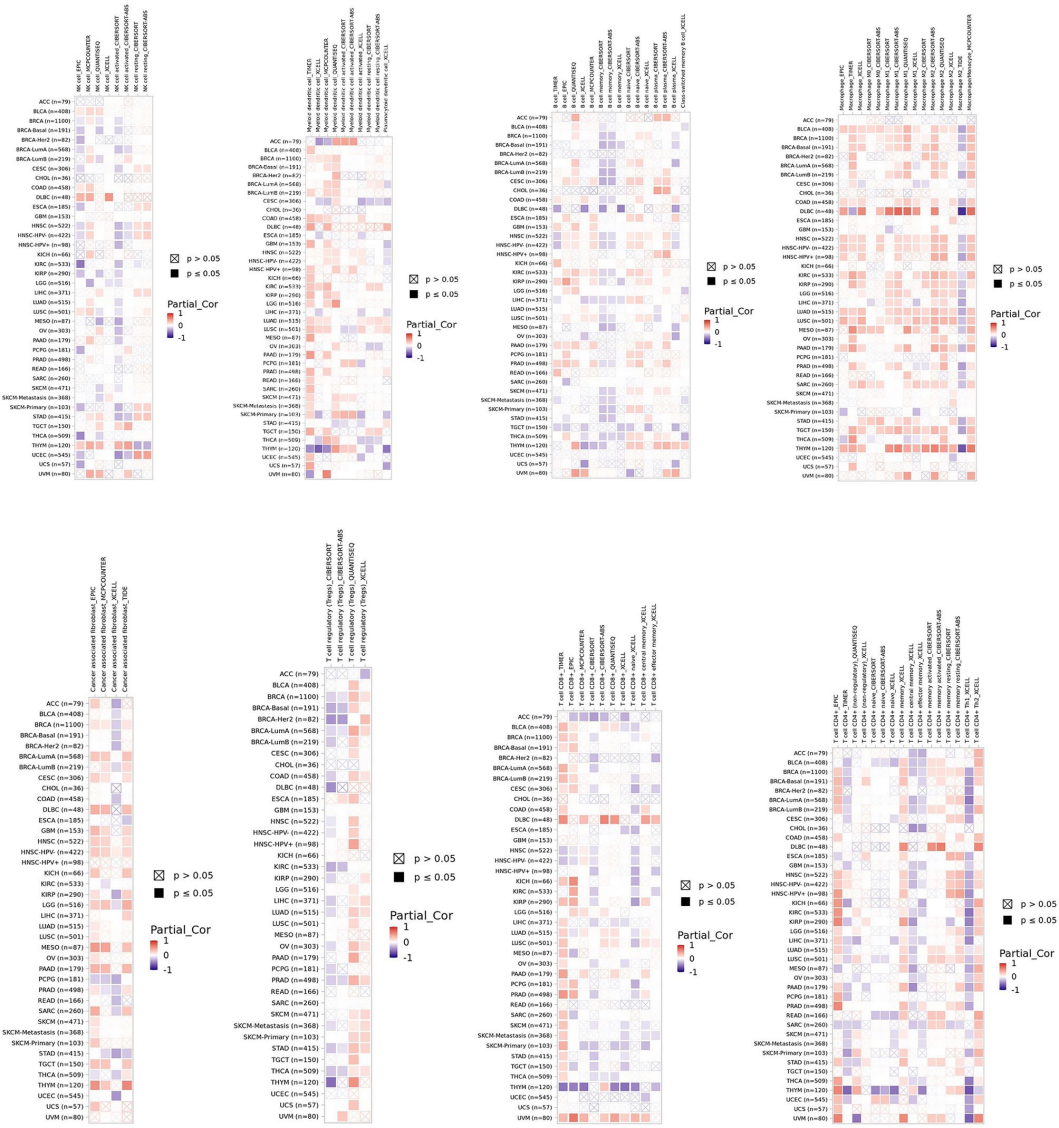
The relationship between SLC31A1 expression and immune cells infiltration. Immune cells, including NK cell, Myeloid Dendritic cell, B cells, Macrophages, Cancer associated fibroblasts, Treg cells, CD8 + T cells and CD4 + T cells. Positive correlation (0–1) are indicated with the red color, while negative correlation (− 1 to 0) are indicated with the blue color. p-value < 0.05 is considered as statistically significant. A cross indicates non-significant correlations. TIMER2.0 was used (Version: 2.0 and link: http://timer.cistrome.org/).

Recent studies have revealed that immune cells, specifically T cells, B cells, and tumour-associated macrophages, play important roles in tumour progression^11–13^. Several algorithms, including TIMER, XCELL, QUANTISEQ, CIBERSORT, CIBERSORT-ABS, EPIC, and TIDE, have been employed to investigate the relationship between SLC31A1 and immune cell infiltration in pan-cancer using TIMER 2.0^5^. For example, in DLBC, a higher expression of SLC31A1 was observed in M1 macrophages, CD8 + T cells, CD4 + T cells, and NK cells. Several studies have demonstrated that M1 macrophages contribute to good tumour outcomes. T-cells, including CD4^+^, CD8^+^, and NK cells, also play an essential role in resisting tumour development. As for other tumour types, some immune cells were negatively correlated with SLC31A1 expression (Fig. 6). SLC31A1 expression was positively correlated with cancer-associated fibroblasts (CAF) in CESC. CAF negatively correlated with SLC31A1 expression in OV. SLC31A1 plays an important role in tumour development in immune cells and may serve as a novel biomarker for immune cell infiltration, as indicated by the results presented above.

### Function analysis of SLC3A1 at single-cell levels

**Figure 7:**
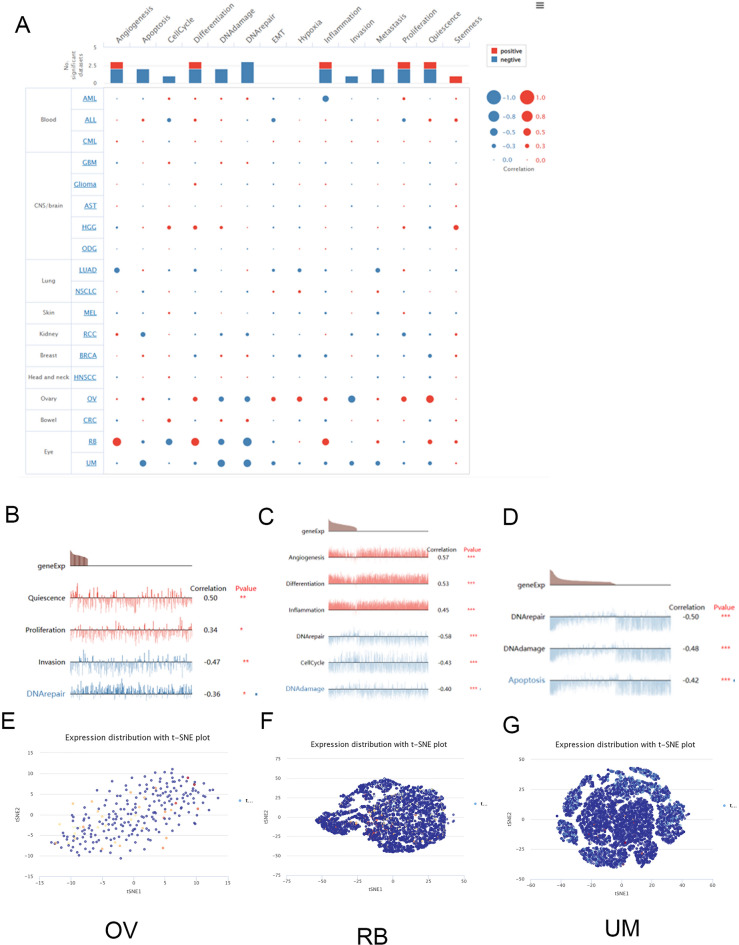
Single-cell function analysis of SLC31A1 in Cancer SEA database. (**A**) Different functional status of SLC31A1 expression in pan-cancer. (**B**–**G**) Correlation analysis between functional status and SLC31A1 and T-SNE diagram in OV (**B**, **E**), in RB (**C**, **F**) and in UM (**D**, **G**). (* p < 0.05, ** p < 0.01, *** p < 0.001).

Recently, single-cell transcriptome sequencing has become one of the primary methodologies for analysing tumour development revealing potential molecular mechanisms^14,15^. Therefore, we utilised The Cancer Single-Cell State Atlas (CancerSEA)^16^ to analyse the correlation between SLC31A1 expression and single-cell biological functions. Figure 7A shows a positive correlation between SLC31A1 expression and angiogenesis, differentiation, and inflammation in RB. SLC31A1 was also positively correlated with DNA repair, DNA damage, and the cell cycle, suggesting that SLC31A1 plays a crucial role in OV development. The positive correlation between SLC31A1 expression and quiescence, proliferation, and hypoxia suggests that SLC31A1 functions as a proto-oncogene in OV. However, additional clinical data are required to validate this molecular mechanism. Moreover, in uveal melanoma (UM), SLC31A1 expression is negatively correlated with most functions, including DNA repair, DNA damage, and apoptosis. In addition, Fig. 7B shows the correlation between SLC31A1 expression and quiescence, proliferation, invasion, and DNA repair in OV. Correlations with angiogenesis, differentiation, inflammation, DNA repair, cell cycle, and DNA damage are depicted in Fig. 7C. Moreover, Fig. 7D shows the same outcomes in UM. T-SNE diagrams also displayed SLC31A1 expression profiles at the single-cell level in OV, RB, and UM (Fig. 7E–G).

### Functional enrichment analysis of SLC31A1 related genes in cancers

**Figure 8:**
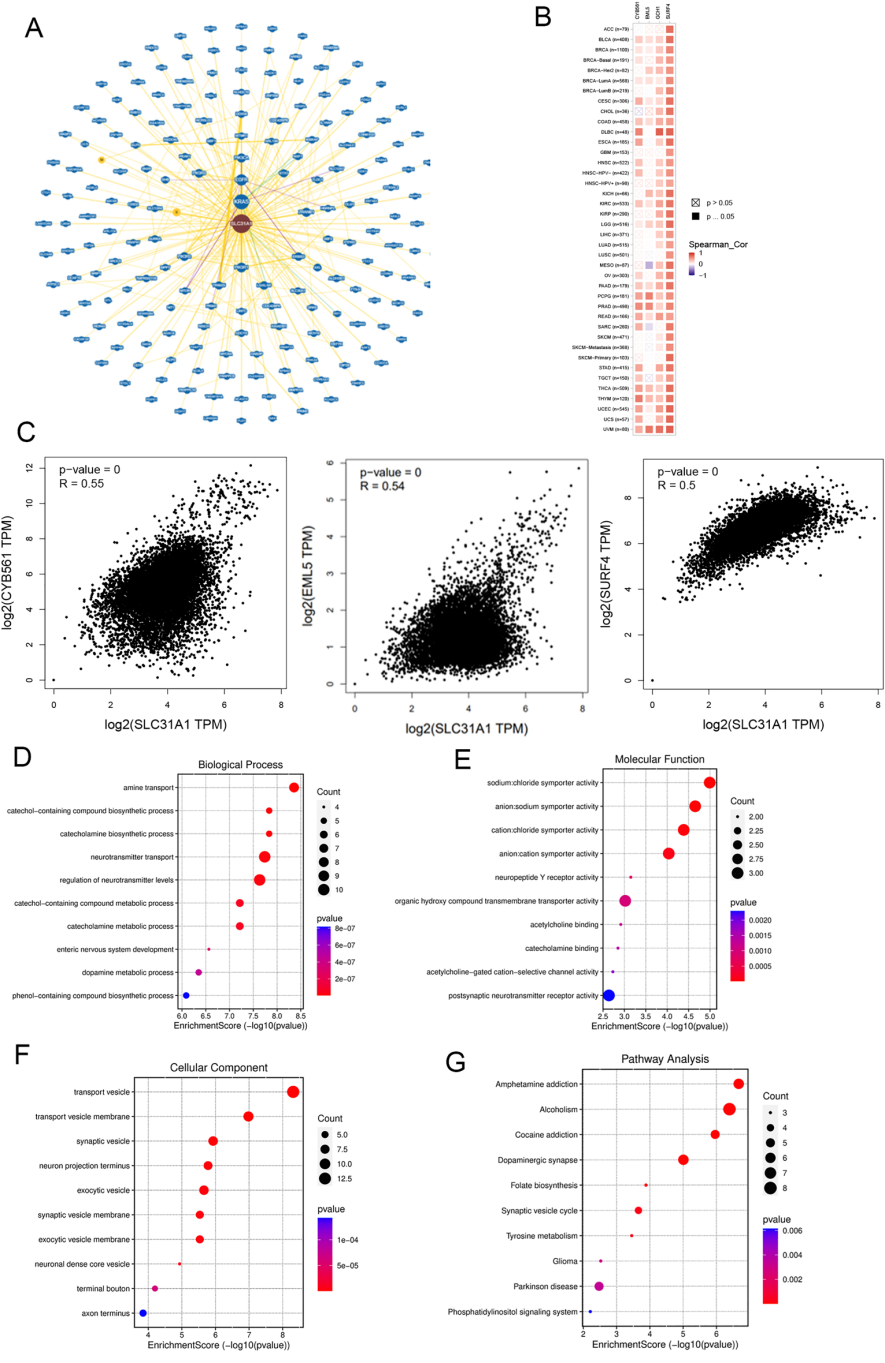
Function enrichment analysis of SLC31A1 related genes. (**A**) The PPI network of SLC31A1 related genes in BioGRID database. (**B**) The positive correlations between SLC31A1 and 3 genes (CYB561, URF4, and EML5) in GEPIA2.0. (**C**) The heatmap of correlation between SLC31A1 and related genes. (**D**–**F**) GO enrichment analysis of SLC31A1 genes in BP (**D**), MF (**E**) and CC (**F**). (**G**) KEGG enrichment of SLC31A1 in pathways.

**Figure 9:**
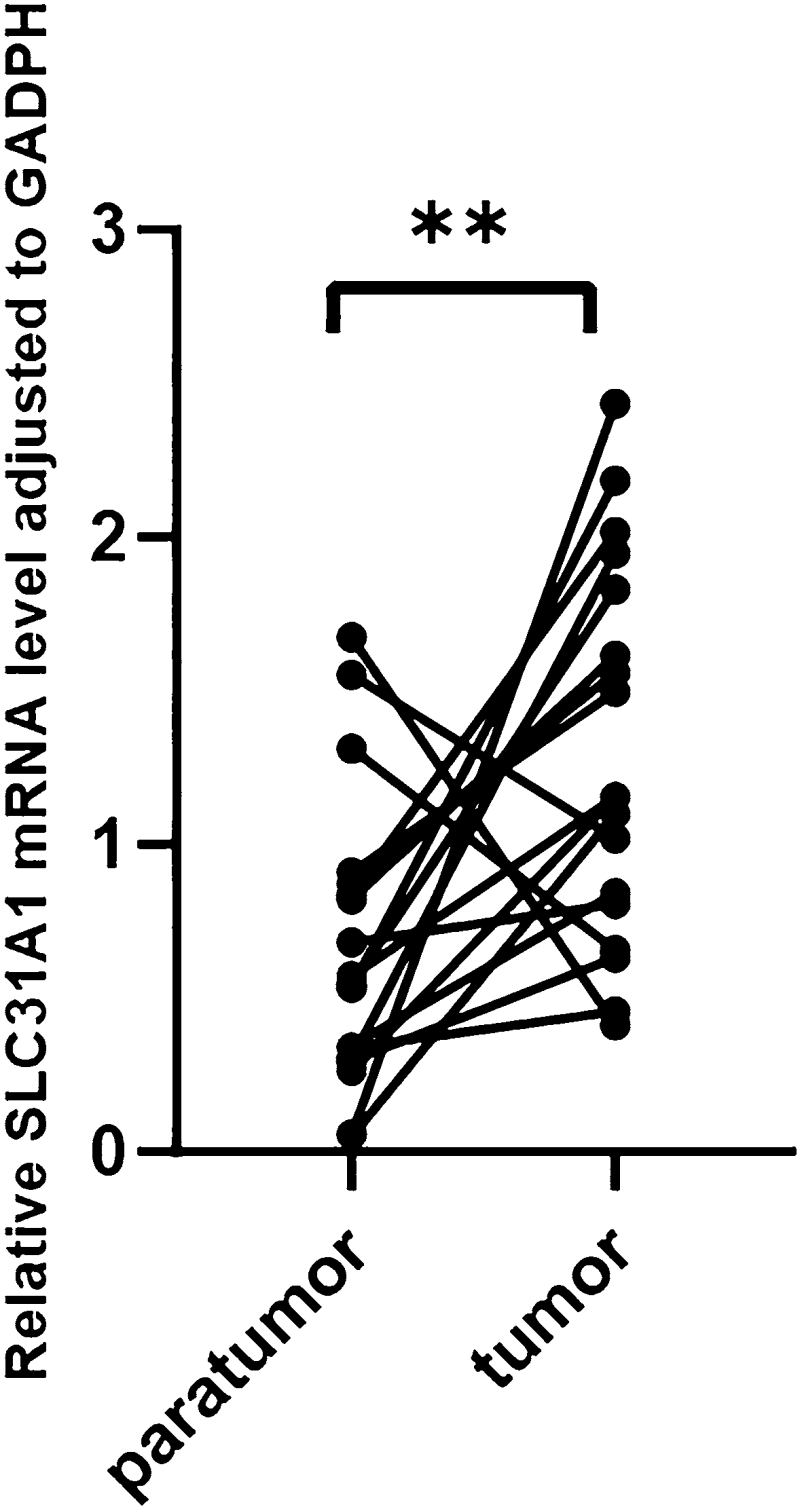
The relative SLC31A1 mRNA level adjusted to GADPH by RT-qPCR. (** p < 0.01).

To investigate the role of SLC31A1 in cancer development, we analysed the molecular mechanisms and protein–protein interactions (PPI) using the Biological General Repository for Interaction Datasets (BioGRID)^17^ and GEPIA2.0. As shown in Fig. 8A, SLC31A1 interacted with 201 genes, of which KRAS and EGFR were the most significantly correlated genes. In addition, we acquired the top 100 SLC31A1 co-expressed genes (Supplementary Table S1) in pan-cancer using GEPIA2.0. While assessing the relationship between SLC31A1 and these genes, we discovered that CYB561, URF4, and EML5 were significantly correlated with SLC31A1 expression in the preponderance of cancers (Fig. 8B, C). Gene Ontology (GO) enrichment, including biological processes (BP), cell components (CC), and molecular functions (MF), was analysed concurrently. The results show that transport vesicles, chloride symporter activity, and neurotransmitter transport-regulated tumour development and tumorigenesis via SLC31A1-associated differentially expressed genes (DEGs) (Fig. 8D–F). Figure 8G reveals that SLC31A1 may play a role via the dopaminergic synapse and synaptic vesicle cycle, based on further analysis of the Kyoto Encyclopaedia of Genes and Genomes (KEGG) pathway enrichment analysis^18,19^. To confirm the expression of SLC31A1 in UCEC, we compared eighteen clinical tissues, including tumour and paratumour tissues, from patients with UCEC. We performed RT-qPCR and found higher SLC31A1 mRNA expression levels in tumour tissues than in normal paratumour tissues (Fig. 9). Based on our findings, we concluded that SLC31A1 is overexpressed in UCEC and has the highest mutation rate. Therefore, SLC31A1 may serve as a potential prognostic biomarker for patients with UCEC. Our study was limited by the fact that more tissue samples are required to investigate the molecular mechanisms of UCEC, however.

## Discussion

Copper is one of the most fundamental ions in the human body, and its dynamic equilibrium is crucial for human homeostasis^20^. Therefore, copper concentration is a double-edged sword. The copper concentration in cells may regulate tumour proliferation and specific enzymes that can induce proangiogenic responses and activate metabolism^21^, but anomalous intracellular accumulation can also result in a novel mode of cell death called cuproptosis^4^. Copper homeostasis was recently found to be significantly associated with gastric cancer^22^, pancreatic cancer^23^, and other tumours^24^, and is governed by protein misfolding^25^, protein lipoylation^4^, and DNA damage response^26^. Consequently, the objective of our study was to examine the key genes associated with copper homeostasis and determine their effects on various tumours.

SLC31A1, also known as CTR1, is a transmembrane protein capable of regulating copper homeostasis. Recent studies have demonstrated that SLC31A1 modulates the cisplatin concentration in multiple malignancies and influences patient prognosis^27–29^. In addition, multiple studies have confirmed a significant negative correlation between SLC31A1 expression and cisplatin resistance^30^. Specifically, overexpression of SLC31A1 in HeLa cells could contribute to a two-fold accumulation of cisplatin^31^. In addition, previous studies have demonstrated that ZNF711 downregulation promoted cisplatin resistance in epithelial ovarian cancer by inhibiting SLC31A1 expression^32^. Further studies have determined the relationship between SLC31A1 and cisplatin chemoresistance in breast cancer, which may serve as prospective prognostic indicator^33^; therefore, SLC31A1 may play a significant role in gynaecological tumours and breast cancer. This is consistent with our finding that SLC31A1 is expressed at higher levels than in normal tissues in CESE and UCEC. In addition, the high SLC31A1 level group had a poor prognosis for patients with BRCA. Therefore, SLC31A1 may be a potential prognostic biomarker, particularly for female patients.

Recent reports indicated that SLC31A1 plays a role in tumour development and is differentially expressed in tumour and normal tissues^34^. Using databases such as TCGA, GTEx, UALCAN, and cBioportal, we analysed SLC31A1 expression in 33 malignancies, and correlations with gene expression, prognosis, gene alterations, immune infiltration, and DNA methylation. Our study confirmed the overexpression of SLC31A1 in 14 tumours, including CESC, UCEC, and BRCA, and the downregulation of SLC31A1 in nine tumours. In addition, our results confirmed a significant correlation between SLC31A1 expression and ACC, MESO, and KIRC in terms of OS and DFS. These findings were consistent with those of previous studies.

Gene mutations are closely associated with tumorigenesis, metastasis, disease deterioration, and prognosis^35^. In our investigation, SLC31A1 mutations were identified in multiple tumours, including in UCEC. According to our findings, UCEC had the highest mutation rate (nearly 2.5%), whereas ACC had the highest amplification. OV also is characterised by many mutations, amplifications, and profound deletions. Consequently, SLC31A1 mutations may contribute to tumorigenesis and play a significant role in this process. DNA methylation is one of the most important biological epigenetic modifications that plays a significant role not only in the expression of genes but also in their stability^36^. Using the UALCAN online tool, we found that SLC31A1 promoter methylation levels were substantially lower in LUSC and READ tissues than in normal tissues, but higher in UCEC, HNSC, KIRP, LIHC, and PRAD tissues. Recent research has indicated that aberrant DNA methylation may promote tumour development by modulating cell proliferation through apoptosis and senescence^37^.

The growth milieu refers to the tumour microenvironment (TME), which consists of tumour, immune, and stromal cells^38,39^. Recent studies have demonstrated that infiltrating immune cells are essential for tumour progression and immunotherapy^40^. Consequently, it is crucial to investigate the relationship between SLC31A1 and immune cell infiltration in the TME. In our study, we discovered a substantial correlation between the expression of SLC31A1 and infiltrated of CD4 + T, CD8 + T, and B cells. In addition, SLC31A1 expression is significantly associated with NK cells, cancer fibroblasts, and macrophages. These results suggest that SLC31A1 is a potential immunotherapeutic biomarker for multiple cancers and provide a theoretical foundation for clinical treatment. However, the molecular mechanisms underlying the association between SLC31A1 expression and immune cell infiltration require further clinical investigation. Analysis of the transcriptome of a single cell revealed that SLC31A1 was associated with angiogenesis, differentiation, proliferation, and inflammation. In addition, SLC31A1 negatively correlated with invasion, cell cycle progression, DNA repair, and DNA damage, particularly in OV, RB, and UM. Recent studies have indicated that SLC31A1 transmits VEGF-induced H_2_O_2_ signals to stimulate angiogenesis^41^. In contrast, our PPI network results support the hypothesis that SLC31A1 regulates EGFR to promote tumour neovascularisation.

To investigate the interaction with other genes, we identified that CYB561, URF4, and EML5 may be associated with SLC31A1 and analysed correlations. However, the network requires further investigation. Using comprehensive bioinformatics analysis, we evaluated SLC31A1 expression, prognosis, DNA promoter methylation, gene variations, and immune cell infiltration at the pan-cancer level. These results indicate that SLC31A1 may play a crucial role in the development and prognosis of various malignancies, particularly UCEC, CESC, and BRCA. However, there are some limitations to this study. Therefore, additional experiments based on data from publicly accessible databases are required. In addition, the underlying molecular mechanisms must be investigated in depth through additional clinical trials and experiments.

In conclusion, our study showed that the cuproptosis-related gene SLC31A1 plays an essential role in tumour prognosis and immune cell infiltration. Our findings suggest that SLC31A1 may be a biomarker for drug resistance and a novel therapeutic target, particularly in gynaecological malignancies.

## Methods

### Gene expression analysis of SLC31A1 in pan-cancer

Using the TIMER2.0 database, differential gene expression between tumour and normal tissues was investigated. Additionally, the GTEX database was used to investigate several malignancies that lacked normal tissues in TCGA. In our study, 33 tumour expression profiles were identified. The CPTAC database was used to assess SLC31A1 protein expression, while GEPIA2.0 was used to analyse the correlation between SLC31A1 expression and pathological stage. The R package “dplyr” was used to sort raw data. The R packages “limma”, “edgeR”, and “DESeq2” were used to analyse differential gene expression and “ggplot” was used to visualise data. Using the HPA, we performed IHC staining for SLC31A1 in greater detail.

### Prognostic analysis of SLC31A1

GEPIA 2.0 was used to examine the prognosis of 33 tumours obtained from TCGA clinical data, including OS and DFS. The R packages “survival” and “survminer” were used to conduct prognostic analyses. The clinical patients were divided into high SLC31A1 expression, and low SLC31A1 expression groups based on the 50% and 50% cut-off values. Hazard ratios (HRs) were calculated using the Cox PH model.

### Gene mutation analysis of SLC31A1

The cBioPortal (https://www.cbioportal.org/) database was used to analyse SLC31A1 tumour genomic characteristics, including mutation, amplification, and profound deletion. The R package “maftools” was used to analyse the mutation. In addition, the 3D structure of SLC31A1 was presented via cBioPortal. In addition, the clinical prognosis between the modified and unmodified groups was downloaded.

### DNA promoter methylation analysis of SLC31A1

The UALCAN database (http://ualcan.path.uab.edu/) was utilised to investigate the promoter methylation of SLC31A1 in 33 tumours compared to that in primary tumours and normal tissues. The R package “methylkit” was used for analysis DNA promoter methylation.

### Relationship between SLC31A1 expression and immune cells infiltration

The relationship between SLC31A1 expression and immune cells, including CD8 + T cells, CD4 + T cells, B cells, NK cells, tumour-associated fibroblasts, and macrophages, was investigated using TIMER2.0. The R package “immunedeconv” was used to integrate six state-of-the-art algorithms: TIMER, xCell, MCP-counter, CIBERSORT, EPIC, and quanTIseq. A positive correlation between SLC31A1 expression and immune cells was indicated by a Spearman’s correlation coefficient of > 0 and p < 0.05, while a negative correlation was indicated by Spearman’s 0 and p < 0.05.

### Single-cell functional analysis of SLC31A1

CancerSEA was used to evaluate cancer cell functions at the single-cell level of SLC31A1, which consisted of fourteen tumour-related cellular functions of 900 cancer cells from 25 malignancies. In addition, T-SNE diagrams confirmed the expression of SLC31A1 in TCGA. The R package “seurat” was used to analyse the single-cell function.

### Gene enrichment analysis

The protein–protein interaction (PPI) network was analysed using the BioGRID online database. The relationship between SLC31A1 expression and the other 100 most frequently downloaded genes from GEPIA2.0 was subsequently analysed using Pearson’s correlation analysis. The BP, CC, MF, and signalling pathways were estimated using GO and KEGG enrichment analyses in the R package “clusterProfiler.” The significance threshold for GO analysis was set to an adjusted p value of 0.05, and the significance threshold for KEGG analysis was set to p < 0.05.

### RNA extraction and real-time quantitative polymerase chain reaction (RT-qPCR)

At Tianjin Central Hospital Gynecology Obstetrics, samples of human tissue were collected. Ethical committee of Tianjin Central Hospital Gynecology Obstetrics granted approval after obtaining informed consent from all patients or their family members. Using TRIzol reagent, total RNA was extracted and quantified using a NanoDrop spectrophotometer. Using a cDNA reverse transcription reagent, total RNA was reverse-transcribed. In a 7500 real-time PCR system with SYBR green, PCR amplification was performed at 95 °C for 30 s, followed by 50 cycles of 95 °C for 10 s and 60 °C for 34 s. The expression of GAPDH mRNA was used to normalize the expression of the target gene in each sample. Analyzing relative mRNA expression levels using the 2−ΔΔCt method. All primers were synthesized by company Jinweizhi (Tianjin, China). SLC31A1 5′-3′ primers: Forward: GGGGATGAGCTATATGGACTCC; Reverse: TCACCAAACCGGAAAACAGTAG.

### Ethics approval and consent to participate

The study was conducted according to the guidelines of the Declaration of Helsinki, and approved by the Ethics Committee of Tianjin Central Gynecology Obstetrics Hospital. Ethical committee of Tianjin Central Hospital Gynecology Obstetrics granted approval after obtaining informed consent from all patients or their family members.

### Supplementary Information

Supplementary Figure S1.

Supplementary Figure S2.

Supplementary Table S1.

## Data Availability

The datasets analyzed during the current study are available in the TCGA (https://xenabrowser.net/ (accessed on 1 March 2023)), GETx (https://www.genome.gov/(accessed on 1 March 2023)), TIMER2.0 database (http://cistrome.dfci.harvard.edu/TIMER/ (accessed on 5 March 2023)) and HPA (https://www.proteinatlas.org/ (accessed on 8 March 2023)) databases.
