# Supplementary figures and images for: Cuproptosis-related gene SLC31A1: prognosis values and potential biological functions in cancer
Source: Sci Rep. 2023 Oct 18;13:17790. doi: 10.1038/s41598-023-44681-8 (PMC10584849; doi:10.1038/s41598-023-44681-8)

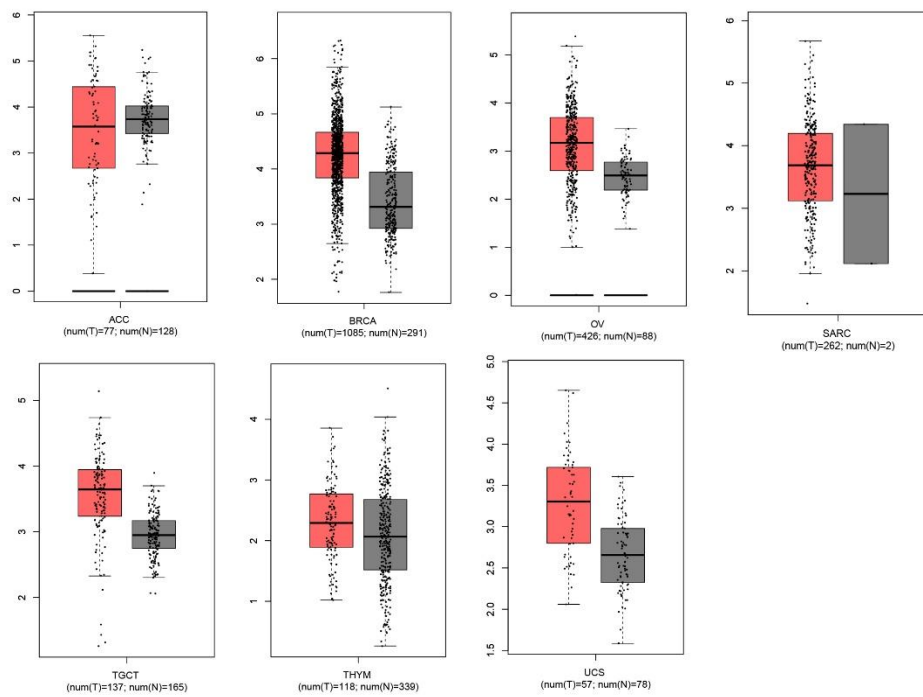

**Figure S1 The expression of SLC31A1 from TCGA and GTEX in ACC, BRCA, OV, SARC, TGCT, THYM and UCS.**

Supplement: Supplementary file 1 — Supplementary Figure S1. [file 41598_2023_44681_MOESM1_ESM.pdf]
