# Supplementary material for: Cuproptosis-related gene SLC31A1: prognosis values and potential biological functions in cancer
Source: Sci Rep. 2023 Oct 18;13:17790. doi: 10.1038/s41598-023-44681-8 (PMC10584849; doi:10.1038/s41598-023-44681-8)
Supplement: Supplementary file 2 — Supplementary Figure S2. [file 41598_2023_44681_MOESM2_ESM.pdf]

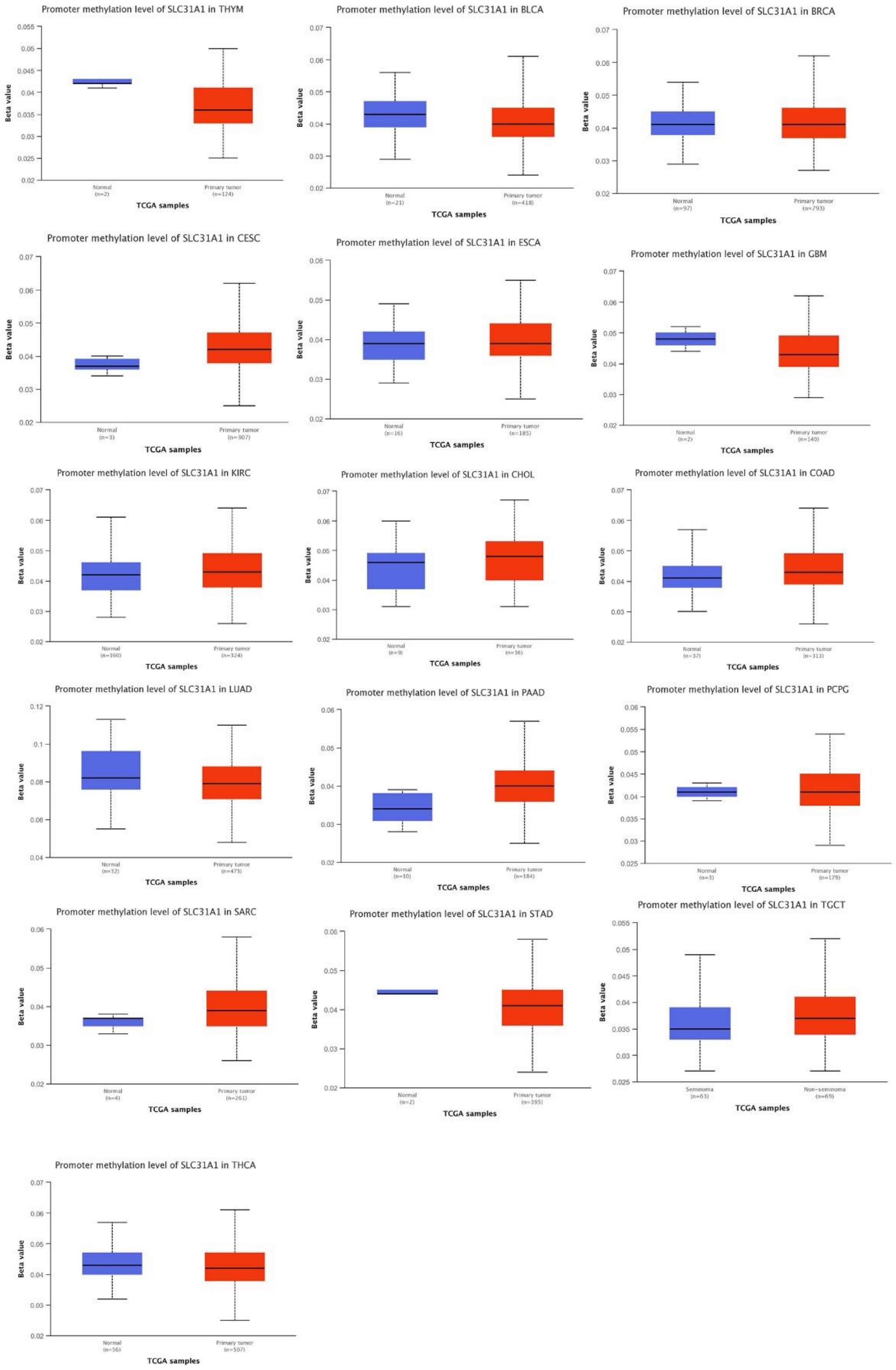

Figure S2 DNA promoter methylation of SLC31A1 in pan-cancer between normal and primary tumor tissues by UALCAN.
